# Altered body representation in chronic non-specific low back pain: a testable framework linking body-schema-related disturbance, corticomotor alterations, and altered pain processing

**DOI:** 10.3389/fpain.2026.1899538

**Published:** 2026-10-08

**Authors:** Zhifei Xie

**Affiliations:** Palmer College of Chiropractic Florida Campus, Port Orange, FL, United States

**Keywords:** body representation, body schema, chronic non-specific low back pain, corticomotor function, nociplastic pain, pain processing, predictive coding, sensorimotor integration

## Abstract

Low back pain is a leading contributor to disability worldwide; in chronic non-specific low back pain (CNSLBP), the processes sustaining pain and disability in the absence of identifiable structural pathology remain incompletely understood. Three partially overlapping research lines have documented altered body representation, corticomotor alterations of trunk musculature, and altered pain processing in this population, but these domains have rarely been measured concurrently within the same cohort. This Hypothesis and Theory article proposes that, once the measurement structure of candidate indicators has been established, body-representation-related measures will provide reproducible incremental explanatory information for prespecified corticomotor and pain-processing outcomes beyond relevant clinical, psychological, and nociplastic-feature variables. Within this multidimensional domain, body-schema-related sensorimotor disturbance is examined as one candidate interacting mechanism that may be coupled to corticomotor and pain-processing abnormalities; it is not assumed to be a privileged or causally primary organizer. Currently available assessments do not directly measure body schema but rely on indirect and theoretically heterogeneous proxies, including self-reported back perception, tactile acuity, and implicit laterality or motor-imagery processing. We synthesize evidence from neuroimaging, transcranial magnetic stimulation, psychophysical pain assessment, and behavioral paradigms, using predictive coding as a heuristic framework for deriving testable predictions concerning trunk-state estimation, motor regulation, and pain-processing indices. The proposed framework suggests that altered trunk-state representation may interact with altered motor regulation and altered pain processing in a mutually reinforcing loop. However, the directional architecture remains untested; models in which altered pain processing, corticomotor alteration, or a shared upstream process contributes to representational disturbance remain viable. The framework specifies a construct-validity prerequisite and four predictions concerning incremental cross-domain association, nociplastic-feature enrichment, intervention-linked cross-domain change, and temporal ordering and model comparison. Current evidence supports several measurable body-representation-related phenomena associated with clinical features in CNSLBP; however, the incremental explanatory value of the broader domain and the mechanistic independence of the narrower body-schema-related component remain empirical questions requiring discriminant, longitudinal, and experimental validation.

## 1 Introduction

Chronic non-specific low back pain (CNSLBP) contributes substantially to global disability, and its burden is projected to increase (1, 2). Approximately 90% of low back pain presentations lack an identifiable pathoanatomical cause (3), while degenerative imaging findings are common in asymptomatic individuals and correlate incompletely with symptom severity (1, 4, 5). These observations limit the adequacy of currently identifiable structural findings as a complete explanation of pain persistence, while leaving open interacting peripheral nociceptive, psychological, social, and central pain-modulatory processes (6). Reviews and studies in chronic low-back-pain and broader chronic-pain populations have reported heterogeneous differences involving sensorimotor and pain-related networks (7–9), but cross-sectional group differences cannot establish whether a given alteration is causal, consequential, compensatory, or confined to a subgroup. A central theoretical problem is therefore to identify measurable processes that may help explain how persistent pain and altered sensorimotor regulation become coupled in particular patients.

Evidence relevant to this problem is distributed across three largely parallel literatures: body-representation-related disturbance (10–13), corticomotor and sensorimotor alterations (14–19), and altered pain processing (20–22). Each contains empirical findings generated using heterogeneous samples, tasks, protocols, and outcomes. The indices grouped under altered body representation sample different phenomena, including self-reported back perception (10, 13), tactile-spatial discrimination (10, 12), and implicit laterality or motor-imagery performance (11). Here, body-perception disturbance refers to conscious perceptual or self-reported phenomena, whereas body-schema-related disturbance denotes the narrower candidate action-oriented sensorimotor component. These indices cannot be assumed to constitute direct or interchangeable measures of a unitary body schema. Because these domains have generally been studied separately, existing evidence does not establish their covariation within the same well-characterized CNSLBP cohort, their temporal ordering, or their comparative explanatory value. The resulting gap is relational and construct-based: whether body-representation-related indices form a reproducible construct, provide information beyond pain intensity, disability, nociplastic-feature indicators, and psychological or behavioral correlates, and participate in cross-domain associations that survive competing-model tests.

We therefore hypothesize that, once the measurement structure of candidate indicators has been established, body-representation-related measures will provide reproducible incremental explanatory information for prespecified corticomotor and pain-processing outcomes beyond relevant clinical, psychological, and nociplastic-feature variables, with stronger associations expected when independently assessed nociplastic features converge. Within this multidimensional domain, body-schema-related sensorimotor disturbance is retained as a focal working hypothesis because its action-oriented formulation permits explicit, conditional predictions linking trunk-state estimation with tactile-spatial processing, motor-imagery performance, corticomotor regulation, and pain-processing outcomes. Its focal status does not imply empirical superiority or causal priority over perceptual, cognitive-affective, interoceptive, behavioral, pain-processing-led, or shared-upstream accounts, which remain viable alternatives. Predictive coding (23) is used as a hypothesis-generating heuristic and has not been empirically adjudicated against competing accounts in CNSLBP. The article develops a measurement-aware framework that compares alternative directional models and specifies construct-validity, longitudinal, experimental, and disconfirmation tests. The causal position and comparative explanatory value of body-schema-related disturbance therefore remain empirical questions.

### 1.1 Literature identification and evidentiary scope

This article is a targeted theoretical synthesis rather than a systematic review. Relevant literature was identified through targeted, non-systematic searches of PubMed/MEDLINE, Scopus, and Web of Science up to August 2026, supplemented by reference-chaining from key empirical studies and recent reviews. Search concepts combined chronic or non-specific low back pain with terms relating to body representation, body schema, body perception, tactile acuity, laterality or motor imagery, transcranial magnetic stimulation or corticomotor control, central sensitization or nociplastic pain, quantitative sensory testing or conditioned pain modulation, and predictive coding or active inference. The purpose was to identify convergent, conflicting, and mechanistically informative evidence relevant to the proposed framework.

Evidence from CNSLBP populations was prioritized. Studies using broader chronic low back pain (CLBP) terminology were also considered when their eligibility criteria were compatible with a triage-based definition of non-specific low back pain or when the population limitation could be stated explicitly. Evidence from specific low back pain subgroups or other chronic pain conditions was used only to inform general mechanistic plausibility and was not treated as direct CNSLBP-specific support. Because this was a targeted, non-systematic synthesis, it does not estimate the prevalence or consistency of the proposed abnormalities across the CNSLBP population.

Within the literature identified through this targeted process, no study has simultaneously measured body-representation-related indices, trunk corticomotor function, and pain-processing measures in the same well-characterized CNSLBP cohort. The construct structure of commonly used body-representation-related indices has not been established through factor-analytic or multitrait–multimethod designs, and the proposed directional relationships have not been tested with path-analytic or longitudinal data. Claims concerning literature gaps are therefore limited to this search process. The framework developed below specifies the measurement architecture, competing models, and disconfirmation conditions required to address these gaps.

## 2 Conceptual background: body representation, body schema, and trunk representation

### 2.1 Multidimensional body representation and the focal body-schema hypothesis

Body representation is used here as an umbrella term for a family of representations of the body implemented across distributed neural systems (24, 25). These representations are partially dissociable (24, 26, 27) but can interact through multisensory and sensorimotor processing (24, 25, 27, 28). The body schema refers here to the action-oriented component of this broader domain (27), which typically operates without conscious monitoring (25), draws on dynamically updated information about bodily posture and the position and movement of body parts (24, 28), and supports action planning and online control (25, 27, 28). Body image refers to perceptual representations of bodily form (24, 27), conceptual knowledge and beliefs about one's body (25, 27), and affective evaluations and attitudes toward it (24, 25, 27). Interoceptive, ownership-related, agency-related, attentional, and affective processes may interact with these representational levels, and the relations among them need not follow a single fixed hierarchy (25, 27). Against this multidimensional architecture, the focal body-schema-related hypothesis is operationalized conditionally. Within the proposed coupling model, if degraded trunk-state estimation is present, functionally coupled to the other measured domains, and adequately represented by the selected indices, a prespecified pattern of coordinated variation should emerge among tactile-spatial, laterality or motor-imagery, and conscious back-perception indicators, together with associations between these indicators and prespecified corticomotor and pain-processing outcomes. This formulation identifies the measurement and association pattern required by the model; it does not equate proxy performance with body-schema dysfunction or assume a causal hierarchy. Perceptual, cognitive-affective, interoceptive, behavioral, pain-processing-led, and shared-upstream processes therefore remain alternative or interacting explanations to be compared empirically.

At the measurement level, no single instrument used in the current literature provides a direct measure of body schema. The left/right judgment task (LRJT) requires participants to make left/right judgments about body-related images and is commonly interpreted as indexing implicit motor imagery, although performance also reflects initial classification and response-related processes (11, 29). For axial judgments, however, the mapping of task performance to body-schema processes is less certain, findings in spinal pain are less consistent than those in limb or facial pain, and the reliability and measurement error of LRJT outcomes in CLBP remain insufficiently established (29, 30). The lumbar two-point discrimination threshold (2PDT) provides a behavioral index of conscious tactile-spatial acuity and may partly reflect somatosensory representational processing, although it does not directly measure cortical organization (12); in CLBP, available evidence supports known-groups validity more clearly than reliability, measurement error, or convergent validity (30). The Fremantle Back Awareness Questionnaire (FreBAQ) assesses self-reported disturbances in back perception (13, 31) and does not directly measure the action-oriented sensorimotor processes attributed here to body schema; its measurement properties remain incompletely established, particularly with respect to content and structural validity, measurement error, and responsiveness (32).

The three measures were selected because the focal hypothesis assigns their outputs to complementary functional positions relative to body-schema-related trunk-state estimation: tactile-spatial information available to the estimate, sampled through lumbar 2PDT (12); action-oriented use of the estimate during covert simulation, sampled through LRJT under embodied-simulation conditions (11, 29); and consciously experienced back perception after integration with attentional, cognitive, and affective influences, sampled through FreBAQ (13). Body-schema-related trunk-state estimation is the proposed common denominator across these sensory, action-oriented, and conscious-report expressions. The framework therefore predicts coordinated, although non-identical, variation across the three measures (23, 33).

The discriminant validity of these measures from one another and from general task performance, pain-related distress, fear, and broader symptom burden remains unresolved (29, 30, 32). In this framework, “body-schema-related disturbance” denotes the candidate sensorimotor component, whereas “body-perception disturbance” is reserved for conscious perceptual or self-reported phenomena. The construct-validity analyses specified in Section 6.5 test whether these measures reflect a common factor, stable and distinguishable subdomains, or largely distinct constructs.

### 2.2 Distributed sensorimotor and multisensory networks supporting body representation

Body-schema-related processing is best understood as a distributed sensorimotor and multisensory function involving primary somatosensory cortex (S1), primary motor cortex (M1), posterior parietal cortex, and premotor–parietal circuits, rather than a single fixed physical cortical map (25, 28). S1 contains somatotopically organized representations of tactile and proprioceptive input that can exhibit experience- and input-dependent changes, including altered response magnitude, excitability, connectivity, or structural associations under persistent pain (9, 12). Recent multivariate functional magnetic resonance imaging findings nevertheless caution against treating CLBP as a simple somatotopic-remapping condition (34). M1, S1, posterior parietal cortex, and premotor circuits jointly contribute to integrating sensory information with action planning and body-state estimation. Accurate motor regulation may depend in part on these distributed estimates, but the observation of network or map differences does not establish that representational disturbance caused the motor alteration. Sustained changes in afferent input or sensorimotor processing could alter body-state estimation, while pain, behavior, and motor adaptation could also drive or maintain the observed neural differences.

### 2.3 The predictive coding framework

Under the predictive coding framework, as formalized by Friston (23), the brain uses hierarchical generative models to predict sensory input, while prediction errors encode mismatches between top-down predictions and bottom-up sensory signals. Within active inference, descending cortical signals are formulated as proprioceptive predictions rather than motor commands; spinal reflex mechanisms act to suppress the resulting proprioceptive prediction errors through movement (23, 33). Applied to the present hypothesis, persistent nociceptive input or altered sensorimotor processing could disrupt the updating of predictions about trunk state, allowing mismatches between predicted and incoming bodily signals to persist. Such persistent mismatches provide a conceptual route by which altered trunk-state estimation could become coupled to changes in motor regulation, bodily perception, and pain processing. The resulting cross-domain pathway is treated here as a testable, model-derived hypothesis; it has not been directly established by the evidence synthesized in this article and must be compared with competing accounts in CNSLBP.

### 2.4 Hypothesized vulnerability of trunk-state estimation

Building on the account above, the model proposes that trunk-state estimation may be particularly vulnerable to disrupted updating under persistent pain or disuse, although this trunk-specific vulnerability has not been directly tested in CNSLBP. Its potential functional relevance can be examined in anticipatory postural control, where trunk-muscle activity must be organized before corrective sensory feedback from predictable perturbations becomes available. In recurrent low back pain, delayed anticipatory activation of transversus. abdominis has been associated with differences in its motor-cortical representation (14); however, that study did not measure trunk-state estimation or establish a body-schema-mediated pathway. The framework therefore predicts that, if degraded trunk-state estimation is present in a subgroup of people with CNSLBP, it will be associated with impaired anticipatory postural regulation and may thereby contribute to functional disability.

### 2.5 Operational definition of CNSLBP in the present synthesis

CNSLBP is commonly defined as low back pain lasting at least 3 months without an identifiable pathoanatomical cause. In clinical practice, this definition is applied through diagnostic triage rather than absolute radiological exclusion. Major clinical reviews and guideline-oriented syntheses define non-specific low back pain operationally through diagnostic triage after exclusion of serious spinal pathology and identifiable radicular or specific causes (1, 3). Within the International Classification of Diseases, 11th Revision, many presentations formerly labeled non-specific low back pain are classified as chronic primary low back pain when they are associated with significant distress or disability and are not better accounted for by another chronic pain diagnosis (35). Requiring complete absence of incidental imaging findings would essentially render CNSLBP a diagnostically empty category: disc degeneration, disc bulging, facet arthropathy, and other age-related imaging findings are common in asymptomatic individuals and increase with age (4, 5), and their presence should therefore be interpreted in the context of the patient's clinical presentation rather than treated as sufficient evidence of pain causation.

Accordingly, in synthesizing the empirical literature for this paper, we treat “CLBP” studies as operationally equivalent to CNSLBP when their inclusion criteria reflect this triage-based approach, i.e., when they explicitly exclude serious spinal pathology and identifiable radicular or structural causes [for example, Kim et al. (12) used Quebec Task Force Classification I–II, excluding nerve root involvement, stenosis, and mechanical instability; Kong et al. (36) recruited explicitly non-specific CLBP]. Studies of populations including specific subgroups, such as failed back surgery syndrome or sciatica [e.g., da Silva et al. (37)] or low-back-related leg pain of discogenic origin [e.g., Pei et al. (38)], are cited with that population caveat made explicit. Studies of non-back chronic pain conditions [orofacial pain in Gustin et al. (39); upper-limb complex regional pain syndrome in Mancini et al. (40)] are similarly cited only with explicit acknowledgment of population mismatch. This operational equivalence reflects the methodological reality of the field, where the absence of a non-specific designation in a study's label does not by itself indicate that the population fails to meet triage-based CNSLBP criteria.

This distinction is particularly important when interpreting the early studies that motivated the present hypothesis. Flor et al. (9) recruited a broad chronic-back-pain sample that included muscular and degenerative diagnoses and therefore provides population-adjacent evidence, whereas Moseley (10), despite using “chronic back pain” in the title, explicitly enrolled participants classified as having non-specific spinal pain after structural and disease factors had been excluded, although the six-person exploratory sample limits generalizability. Subsequent studies help determine how far these early observations can be extended to the CNSLBP population defined above. The triage-compatible cohort studied by Kim et al. (12) and the explicitly non-specific cohort studied by Kong et al. (36) showed related tactile-acuity, S1 structural, and functional-connectivity findings, while Hotz-Boendermaker et al. (41) reported altered secondary somatosensory cortex (S2) responses and lumbar somatotopic organization in non-specific CLBP. Sharma et al. (42) add preliminary S1 neurochemical evidence, but the inclusion of participants with radiating leg pain makes that study less directly representative of CNSLBP. The occurrence of related findings in these more contemporary cohorts supports the relevance of somatosensory alteration to clinically defined CNSLBP. However, the studies assess non-equivalent phenomena and do not directly reproduce Flor et al.'s medial magnetoencephalography source displacement or the association between evoked-response magnitude and pain duration; preserved mechanoreceptive thoracolumbar somatotopy in more recent multivariate functional magnetic resonance imaging further limits a uniform remapping interpretation (34). Accordingly, the evidence reviewed below is treated as evidence of candidate, potentially subgroup-specific somatosensory features within clinically defined CNSLBP, rather than as a neural criterion by which CNSLBP itself is identified.

## 3 Altered body representation in CNSLBP: evidence across candidate indicators

Altered body representation in CNSLBP has been investigated through non-equivalent behavioral, perceptual, self-report, and neurophysiological measures. The following sections examine what each measure captures, what differences have been reported in CNSLBP or closely related low-back-pain populations, and how directly those findings bear on the narrower body-schema-related hypothesis.

### 3.1 Lumbar tactile-spatial acuity

The lumbar two-point discrimination threshold (2PDT) is the minimum separation at which two tactile contacts applied to the lower back are perceived as distinct; higher thresholds indicate poorer tactile-spatial acuity (12, 43). Kim et al. reported higher lumbar, but not finger, 2PDT values in participants with CLBP than in pain-free controls (12). In persistent non-specific low back pain, higher thresholds were found in the subgroup classified as having predominant central-sensitization-related features, whereas the subgroup without those features did not differ from pain-free controls (44). A more recent mixed-duration non-specific low-back-pain study found no significant overall group effect, although a corrected pairwise comparison showed higher 2PDT values among participants experiencing pain on the day of testing than among pain-free controls (43).

The accompanying neural findings were associative. Participants with CLBP showed greater S1 gray-matter volume and lower S1-adjacent white-matter fractional anisotropy than controls, while worse lumbar 2PDT was associated with greater gray-matter volume in the S1 back representation (12). In the more recent study, lumbar 2PDT covaried with contralateral S1–thalamic functional connectivity and S1 mean diffusivity, although the latter relationship differed across groups and none of the investigated magnetic resonance imaging measures showed between-group differences (43). These findings support altered lumbar tactile-spatial processing in some CNSLBP samples and subgroups, with possible variability according to current pain status and sensitization-related features. Together, these findings suggest that elevated lumbar 2PDT may characterize a subset of people with CNSLBP, providing a candidate measure for testing whether tactile-spatial disturbance covaries with the corticomotor and pain-processing domains specified by the framework.

### 3.2 Laterality recognition deficits

Trunk laterality-judgment tasks provide a complementary way to examine action-oriented processing of trunk configuration and movement. Their relevance to the body-schema-related hypothesis rests on the proposal that some task versions elicit covert simulation of one's own trunk moving into the depicted posture (11, 45). Earlier conventional tasks showed reduced accuracy in some back-pain samples (11, 46), although the pooled accuracy estimate was substantially heterogeneous (29) and a later CNSLBP study found unchanged behavioral performance accompanied by greater occipital N1 amplitude (47). By contrast, large-amplitude whole-body tasks designed to strengthen person-centered simulation produced longer response times with preserved accuracy (45, 48). Comparison-group definition may also matter: in a larger chronic non-specific low-back-pain cohort, slower and less accurate judgments were observed relative to pain-free participants without previous low back pain, but not relative to pain-free participants with such a history (49). These patterns motivate a task-conditional hypothesis: if LRJT performance reflects altered trunk-state representation, differences should be strongest under conditions that require embodied simulation and should be selective for trunk rather than non-painful control regions. Effects better explained by general visual or attentional demands would weaken its contribution to the proposed body-schema-related pathway, while pain history should be tested as a potential modifier.

### 3.3 Self-reported body perception disturbance

At the conscious self-report level of altered body representation, FreBAQ captures disturbances in back perception, including a reduced sense of connection to or control over the back and uncertainty about its position, outline, size, or symmetry (13). Across chronic non-specific low back pain studies, FreBAQ scores have generally been higher than in pain-free comparison groups (32). In an axial CLBP cohort, higher scores were associated with greater pain and disability and with pain catastrophizing, fear-avoidance beliefs, and psychological distress; modest independent associations with pain and disability remained after adjustment (13). Because content and structural validity remain incompletely established, FreBAQ is used here as a candidate observed indicator of conscious back-perception disturbance rather than as a validated unitary measure of body schema (32). Its role in the framework is to test whether this self-reported component contributes information beyond pain, disability, and psychological distress and shows prespecified associations with tactile-spatial acuity, laterality-judgment performance, corticomotor regulation, and pain-processing measures.

### 3.4 Perceived back form and trunk-movement perception

Beyond questionnaire-based reports, body-drawing and virtual-mirror paradigms provide task-based observations of perceived back form and trunk movement. In a selected clinical sample of six people with chronic non-specific back pain, all produced disrupted drawings of their perceived back outline, whereas none of ten comparison participants without recent back pain did; five patients were unable to delineate part of the trunk, and in each case the poorly delineated region coincided spatially with locally elevated 2PDT (10). This correspondence provides a preliminary basis for testing whether conscious back localization and tactile-spatial acuity vary together. A virtual-mirror paradigm examined movement perception by requiring 15 male military personnel with CNSLBP and 15 military controls to compare their actual trunk flexion with scaled avatar feedback (50). The CNSLBP group tended to overestimate its own trunk movement, but the between-group difference in point of subjective equivalence did not reach conventional statistical significance (*p* = 0.061), and the groups discriminated movement scaling similarly (50). Together, these paradigms extend the assessment of altered body representation beyond self-report by probing two complementary aspects of back perception: the perceived spatial form of the back and the calibration of trunk movement against visual feedback.

### 3.5 Neural correlates and central-sensitization-related features

Several of the body-representation-related measures described above have also been examined alongside neural or sensitization-related features. At the tactile-spatial level, higher lumbar 2PDT thresholds were associated with greater gray-matter volume in the corresponding S1 back representation (12). This region-specific association provides a candidate neural link between a body-representation-related behavioral measure and the somatosensory processing of signals from the back. Its relevance to the narrower body-schema hypothesis depends on whether tactile-spatial performance also shows prespecified associations with action-oriented and motor-regulatory measures.

Clinical heterogeneity may also shape the distribution of these findings. Lumbar 2PDT was elevated in participants classified as having predominant central-sensitization-related features (44), and nine-item Central Sensitization Inventory symptom burden was associated with FreBAQ scores in a mixed-duration outpatient low-back-pain sample (51). Together, these observations identify sensitization-related heterogeneity as a potentially relevant context for variation in tactile-spatial and conscious back-perception findings across low-back-pain samples and subgroups.

### 3.6 Synthesis

Taken together, the evidence reviewed in this section indicates that altered processing of back-related information can be expressed across tactile-spatial, perceptual, self-reported, and action-oriented levels in at least some people with CNSLBP. Associations with S1 structure or connectivity (12, 43) and with sensitization-related features (44, 51) provide initial links between these observations and broader neural and clinical variation. This evidence provides an empirical basis for treating altered body representation as a potentially clinically relevant, multidimensional domain.

Within this broader domain, the body-schema-related hypothesis specifies one narrower sensorimotor possibility: disturbance in action-oriented trunk-state estimation may contribute to a structured pattern across tactile-spatial processing, laterality or motor-imagery performance, conscious back perception, and trunk-movement perception. The framework treats the coherence, incremental value, and causal status of this proposed pattern as separate empirical questions to be evaluated through prespecified relationships with motor and pain-processing domains. The next section examines corticomotor organization and sensorimotor integration as the motor-system domain relevant to that test.

## 4 Corticomotor alterations and sensorimotor integration in CNSLBP

### 4.1 Corticomotor excitability and anatomical specificity

Within the motor domain, transcranial magnetic stimulation (TMS) studies suggest that corticomotor excitability differences in CLBP may extend beyond cortical representations of the trunk. In a predominantly non-specific CLBP cohort, stimulation of the hand area of M1 and recording from the first dorsal interosseous showed a higher resting motor threshold and lower intracortical facilitation, whereas motor-evoked potential amplitude and short-interval intracortical inhibition were unchanged (18). A separate non-specific CLBP cohort also showed a higher resting motor threshold and lower motor-evoked potential amplitude recorded from the first dorsal interosseous (19). Corticomotor profiles varied further across non-specific low back pain, sciatica, and failed-back-surgery syndrome, with the most pronounced abnormalities occurring in the failed-back-surgery group (37). These findings establish remote-muscle excitability as a measurable motor-domain comparator and show why clinical phenotype must be considered when interpreting the observed pattern. Within the framework, a trunk-selective pattern of association would be more consistent with the focal sensorimotor hypothesis, whereas comparable changes in a remote hand muscle would increase the plausibility of a generalized motor-system state or shared-upstream influence. Trunk-specific cortical organization and its relationship with motor control are considered next.

### 4.2 Trunk-muscle M1 organization and motor control

Trunk-muscle TMS studies are particularly relevant to the proposed framework because they localize motor-system variation to the body region implicated in the trunk-state hypothesis and, in some studies, relate cortical organization to observable motor regulation. In recurrent non-specific low back pain, the transversus. abdominis representation was located more posteriorly and laterally and showed greater map volume; both map features were associated with delayed anticipatory activation of the muscle (14). A separate study reported reduced map volume and loss of spatial separation between deep multifidus and longissimus erector spinae representations (15). Later neuronavigated mapping identified muscle-specific center-of-gravity differences but little overall correspondence between map organization and sensory or motor performance (52). Exploratory comparisons across persistent-low-back-pain subgroups also suggested that interindividual map variability and selected measures of overlap may differ across presumed pain mechanisms (53).

Motor regulation also depends on intracortical circuitry and its relationship with voluntary muscle activation. In lateralized CLBP, Massé-Alarie et al. found a hemisphere-specific reduction in short-interval intracortical inhibition and an absence of the relationship between active motor threshold and multifidus activation observed in pain-free controls, while most other excitability and activation measures were comparable (16). Because low-back-muscle TMS responses reflect bilateral descending projections and are sensitive to stimulation and recording protocols, individual map and excitability outcomes require measure-specific interpretation (54, 55). For the present framework, trunk-muscle M1 organization and motor regulation therefore constitute the motor-domain outcomes against which body-representation-related measures can be tested. Their functional expression also depends on how lumbosacral afferent input modulates corticospinal output.

### 4.3 Afferent modulation of trunk corticospinal output

Massé-Alarie et al. examined this afferent–motor interface by conditioning paravertebral TMS responses with non-noxious cutaneous, noxious cutaneous, and muscle-related stimulation of the lumbosacral region (17). The ratio of conditioned motor-evoked potential amplitude to electromyographic activity in the lumbar multifidus was greater in CLBP than in pain-free controls at 60, 80, and 100 ms, a pattern interpreted as greater facilitation of descending corticospinal input after accounting for net motoneuronal excitability (17). The group difference was expressed across stimulation types and therefore represents a general afferent-conditioned response; nociceptive specificity remains unresolved (17). This sensory-to-motor transformation is theoretically relevant because an action-oriented estimate of trunk state could influence motor regulation at this interface. The magnitude and timing of the conditioned response therefore provide motor-domain outcomes for concurrent tests of the proposed relationship with body-representation-related indices.

### 4.4 Synthesis

The evidence reviewed in this section defines the motor-system domain of the framework at three analytically distinct levels: remote-muscle corticospinal and intracortical excitability, trunk-muscle M1 organization and motor regulation, and lumbosacral afferent modulation of trunk corticospinal output. Remote-muscle measures provide a comparison for a more generalized motor-system state, whereas the latter two levels characterize motor organization and sensory-to-motor regulation more directly related to the trunk. Variation across measures, target muscles, protocols, and clinical presentations should therefore be represented explicitly rather than collapsed into a single corticomotor abnormality. A recent systematic review and meta-analysis of 33 studies provides the strongest current quantitative synthesis of this motor domain (56). It found evidence of low to moderate certainty for lower paraspinal corticospinal excitability, with the most robust pooled findings involving motor-evoked potential amplitude at 120% of active motor threshold and cortical map volume. Apparent differences in cortical map area for paraspinal and abdominal muscles did not remain significant in sensitivity analyses, while evidence concerning hand muscle resting motor threshold and abdominal map location was of very low certainty. These results support separate interpretation by target muscle and outcome rather than a unitary corticomotor abnormality. Within the framework, retaining these outcomes separately allows relationships with body-representation-related measures to be examined as trunk-focused, broadly distributed, or specific to a particular motor process. The afferent-conditioned response remains a motor-system outcome because it quantifies modulation of trunk corticospinal output; pain-processing measures constitute the separate third empirical domain developed in Section 5.

## 5 Altered pain processing within the proposed framework

Altered pain processing constitutes the third empirical domain of the framework. It is operationalized most directly through psychophysical measures of mechanical pain sensitivity, pronociceptive facilitation, and endogenous pain modulation (57, 58). Neuroimaging and neurophysiological findings are treated separately as candidate correlates, substrates, or temporal predictors because changes in cortical structure, connectivity, or excitability do not themselves establish altered nociceptive processing. The framework therefore allows pain-processing alterations to precede, follow, or interact reciprocally with body-representation and motor-system changes.

### 5.1 Psychophysical dimensions of altered pain processing

In the present framework, altered pain processing is operationalized across three complementary psychophysical dimensions: mechanical pain sensitivity, pronociceptive facilitation, and endogenous pain modulation. Pressure pain thresholds assess local or widespread mechanical sensitivity (57), temporal summation of pain (TSP) captures the increase in perceived pain during repeated noxious stimulation (58), and conditioned pain modulation (CPM) assesses the change in a test-pain response produced by a separate conditioning stimulus (59). These measures provide directly observed pain-processing variables for comparison with body-representation and corticomotor measures. Because they capture different aspects of pain processing, they are retained as separate variables rather than combined *a priori*.

Lower pressure pain thresholds at remote sites have been reported in non-specific low back pain, consistent with mechanical hypersensitivity extending beyond the symptomatic region in some samples (57). Facilitated TSP has been observed in acute or recurrent low back pain as well as in CLBP (58), whereas CPM shows a different chronicity pattern. Meta-analytic evidence indicates less efficient modulation in CLBP, whereas a comparable difference was not detected in acute or recurrent low back pain (58). A CLBP-specific systematic review also reported mixed CPM findings across studies and testing protocols (59). Within CLBP, CPM and TSP were only modestly associated after adjustment for demographic variables and negative affect, suggesting that inhibitory and facilitatory responses do not necessarily vary in parallel (60). Pressure pain thresholds, TSP, and CPM are pain-processing measures and carry no direct body-schema interpretation. Within the framework, these measures operationalize complementary dimensions of a separately measured pain-processing domain against which body-representation-related and corticomotor measures can be compared. Their cross-domain associations, temporal ordering, and intervention-linked changes provide the observations required to test incremental association and to compare body-representation-led, motor-system-led, shared-upstream, and pain-processing-led accounts.

Interpreting these psychophysical dimensions also requires distinguishing them from broader clinical and mechanistic terms. Nociplastic pain is a mechanistic descriptor operationalized through graded clinical criteria that include chronicity, pain distribution, insufficient explanation by nociceptive or neuropathic mechanisms, and clinically evoked pain hypersensitivity (61). Central sensitization denotes increased responsiveness of nociceptive neurons in the central nervous system (62). The Central Sensitization Inventory is a patient-reported measure of symptoms associated with central sensitivity syndromes (63) and cannot by itself establish central sensitization or nociplastic pain (62). Clinical criteria, symptom burden, and psychophysical responses are therefore treated as related but non-interchangeable sources of information. Patterns of convergence across these sources can characterize the pain-processing context in which cross-domain relationships are examined. This measurement hierarchy provides the basis for interpreting the neural-system findings considered next.

### 5.2 Somatosensory cortical correlates of back-related processing

The psychophysical measures described in Section 5.1 define the pain-processing phenotype, whereas somatosensory cortical studies provide a separate neural level at which painful and nonpainful back-related input can be examined. In a foundational magnetoencephalography study of a small sample selected using older, broad chronic-back-pain criteria, painful back stimulation elicited greater early evoked-response power and a more medial estimated S1 activity maximum in the very chronic subgroup; response power increased with pain duration (9). Using nonpainful mechanical stimulation of individual lumbar vertebrae, Hotz-Boendermaker et al. found no S1 reorganization but observed reduced S2 activation extent and less differentiated lumbar somatotopy in CLBP (41). These findings indicate that cortical processing of back-related input may differ across sensory conditions and cortical levels, motivating separate consideration of response magnitude and spatial organization within the framework.

A measurement-level bridge to the body-representation domain comes from the relationship between lumbar tactile acuity and S1 structure. Kim et al. linked worse lumbar 2PDT to greater gray-matter volume in the S1 back representation, alongside lower fractional anisotropy in adjacent white matter (12). This association identifies a specific cross-domain question: whether S1 structural variation covaries independently with tactile-spatial and psychophysical pain-processing measures when they are assessed in the same cohort. In a small and clinically mixed CLBP sample, preliminary proton magnetic resonance spectroscopy findings further suggested that S1 metabolite profiles may vary with pain duration or severity (42). Structural and neurochemical differences are therefore treated as candidate local cortical correlates whose relationships with body-representation and pain-processing measures require direct testing. These local cortical correlates raise the further question of how back-related somatosensory variation is situated within broader functional-network organization.

### 5.3 Functional connectivity and distributed network organization

Functional-connectivity findings extend the analysis from local cortical features to the distributed systems in which back-related signals are processed. In non-specific CLBP, S1/M1-centered whole-brain hub connectivity was lower than in pain-free controls during a lower-pain condition and increased within patients when endogenous back pain was exacerbated (36). Across CLBP imaging studies, resting-state connectivity and pain-evoked activity differences have also involved sensorimotor, default-mode, prefrontal, cingulate, insular, and amygdalar systems (8). These observations place local S1/S2 differences within a broader network context that varies with pain state and encompasses sensory, motor, attentional, and affective functions.

Within the proposed framework, this network context creates two distinguishable interpretations of cross-domain association. A selective relationship between body-representation-related measures and back-related somatosensory connectivity would be compatible with a focused sensorimotor link. Parallel relationships of body-representation, corticomotor, and psychophysical pain-processing measures with distributed network features would increase the plausibility of a broader pain-state or shared-upstream account. Existing studies have not measured these domains concurrently, leaving these alternatives unresolved. These network-level relationships leave open whether the spatial organization of back-related cortical representations is itself altered.

### 5.4 Somatotopic organization and the scope of the framework

The early magnetoencephalographic finding concerned the location and magnitude of S1 responses to painful back stimulation (9), whereas recent multivariate functional magnetic resonance imaging examined the segmental organization of non-noxious mechanoreceptive thoracolumbar input (34). The latter study identified organized representations across S1, M1, and superior parietal cortex in both CLBP and pain-free participants, without corrected group differences or associations between representational organization and pain measures (34). Because the studies differ in stimulus modality and in the representational property analyzed, the later finding is not a direct replication of the earlier pain-evoked result. It nevertheless weighs against spatially degraded mechanoreceptive back maps as a necessary feature of CLBP, while leaving nociceptive somatotopy and mechanism-defined subgroup effects unresolved.

Importantly for the present framework, participants with CLBP nevertheless reported greater conscious back-perception disturbance on the FreBAQ, while FreBAQ scores were not associated with mechanoreceptive representational organization (34). Conscious back-perception disturbance and degradation of the mechanoreceptive cortical map therefore cannot be treated as interchangeable phenomena. Somatotopic organization is retained as a separately testable neural feature rather than used as a proxy for altered body representation or body-schema-related disturbance. Support for the broader hypothesis must instead depend on the measurement structure of its candidate body-representation indices and on their prespecified relationships with corticomotor and pain-processing measures. The three-domain model can therefore be evaluated without requiring a universal cortical-remapping mechanism.

### 5.5 Synthesis

In the proposed model, pain processing is operationalized through pressure pain thresholds, temporal summation of pain, and conditioned pain modulation, which capture mechanical sensitivity, pronociceptive facilitation, and endogenous modulation. The cortical findings considered above provide a possible neural context for variation in these psychophysical measures. The pain-processing component of the cross-domain hypothesis is therefore that, once the measurement structure of candidate body-representation indices has been established, these indices should show prespecified associations with psychophysical pain-processing and corticomotor measures in at least a subgroup of people with CNSLBP.

One empirical point of contact with motor control is the modulation of trunk corticospinal output by lumbosacral afferent input. In CLBP, lumbosacral conditioning produced greater facilitation of corticospinal input to lumbar multifidus than in pain-free controls after accounting for net motoneuronal excitability (17). The group effect was not shown to differ among noxious cutaneous, non-noxious cutaneous, and muscle-related conditioning, identifying altered afferent–motor regulation without establishing a nociception-specific pathway. The broader relationship may also operate behaviorally: pain-related fear and avoidance can alter movement behavior (64), while restricted movement may change the bodily information available for updating internal models of the back (65). These directions define a potentially reciprocal relationship in which body-schema-related disturbance is proposed as one possible contributor alongside pain-processing-led, motor-system-led, and shared-upstream processes. Section 6 formalizes this proposition as competing directional architectures and testable predictions.

## 6 Proposed integrative model

### 6.1 Integrative premise

The proposed model links body representation, corticomotor regulation, and pain processing at the level of relationships among domains measured in the same individuals. Figure 1 distinguishes findings already observed within the three domains from the two focal coupling relationships proposed between body-representation-related measures and the corticomotor and pain-processing domains. Because these domains have largely been studied in different samples, their separate occurrence in CNSLBP does not establish how they are related within individuals. The model therefore begins with a conditional cross-domain proposition: once the measurement structure of candidate body-representation indices has been established, these indices should show reproducible, prespecified associations with corticomotor and pain-processing measures in at least a subgroup of people with CNSLBP.

**Figure 1:**
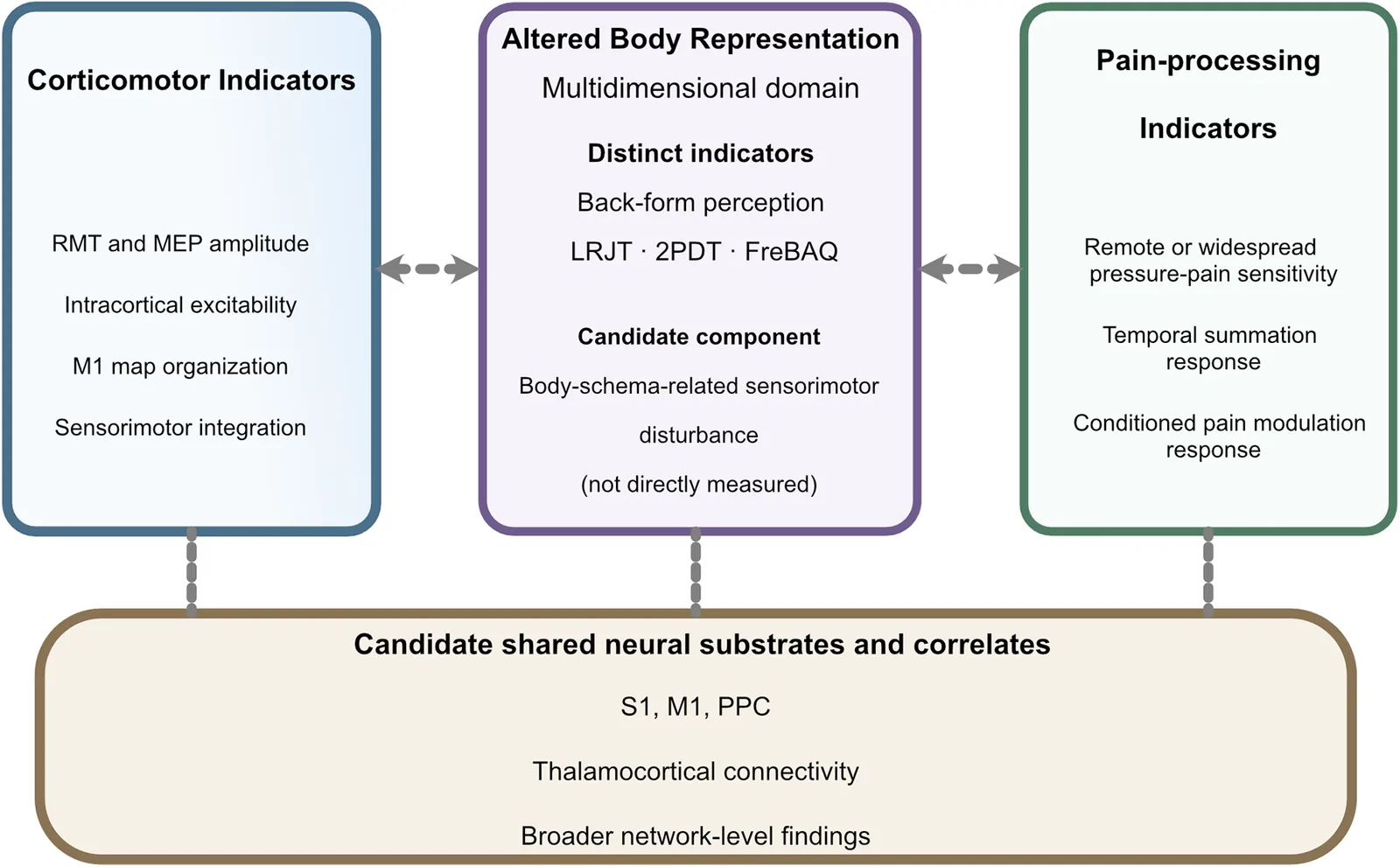
Evidence map of body-representation-related, corticomotor, and pain-processing findings in chronic non-specific low back pain (CNSLBP). Altered body representation is presented as a multidimensional domain: back-form perception, LRJT, 2PDT, and FreBAQ sample distinct, non-interchangeable phenomena, whereas body-schema-related sensorimotor disturbance remains a candidate component not directly measured by any single indicator. The lateral boxes summarize selected corticomotor and pain-processing indicators. Dashed bidirectional arrows indicate possible coupling of body-representation-related measures with corticomotor and pain-processing measures, without implying temporal order, causality, or priority. The lower box lists candidate shared neural substrates and correlates; vertical dashed connectors indicate possible cross-level relevance, not a common mechanism or causal direction. Findings may vary by protocol, outcome, or subgroup. LRJT, left/right judgment task; 2PDT, two-point discrimination threshold; FreBAQ, Fremantle Back Awareness Questionnaire; RMT, resting motor threshold; MEP, motor-evoked potential; M1, primary motor cortex; S1, primary somatosensory cortex; PPC, posterior parietal cortex.

### 6.2 A body-schema-related working model of cross-domain coupling

The working model assigns a candidate organizing role to the body-schema-related information used to estimate trunk configuration during action, because body schema supports movement planning, action prediction, and the use of sensory feedback (27). In the predictive-coding account introduced in Section 2.3, bodily-state estimates are updated according to mismatches between predicted and incoming sensory signals and the precision assigned to those mismatches (23); active inference extends this logic to movement by treating descending motor signals as proprioceptive predictions fulfilled through reflex-mediated action (33). Bringing these premises together, the model proposes that, in a subgroup of people with CNSLBP, an insufficiently calibrated or incompletely updated estimate of trunk state could alter how tactile, proprioceptive, and movement-related information is reconciled with the expected configuration of the trunk. Because that estimate also informs predictions about the sensory consequences of action, the same disturbance could contribute to variation in afferent–corticomotor integration and in anticipatory or voluntary trunk-motor regulation.

The same updating problem could extend to pain processing when trunk-related signals are processed in the context of persistent pain or pain-related threat. Under these conditions, changes in the weighting and interpretation of nociceptive and non-nociceptive bodily information could link variation in trunk-state estimation to psychophysical pain-processing responses. Both pathways remain model-derived in CNSLBP; the motor route follows more directly from the action-oriented role of body schema and active inference, while the pain-processing route is a further extension of those premises. Together, they define the body-representation-led architecture shown in Figure 2A, in which trunk-state estimation is proposed as a candidate contributor to structured associations among body-representation-related, corticomotor, and pain-processing measures in some patients. If cross-domain coupling is present, changes in pain, movement, and sensory sampling may also influence trunk-state updating, providing a possible basis for mutual reinforcement among the three domains.

**Figure 2:**
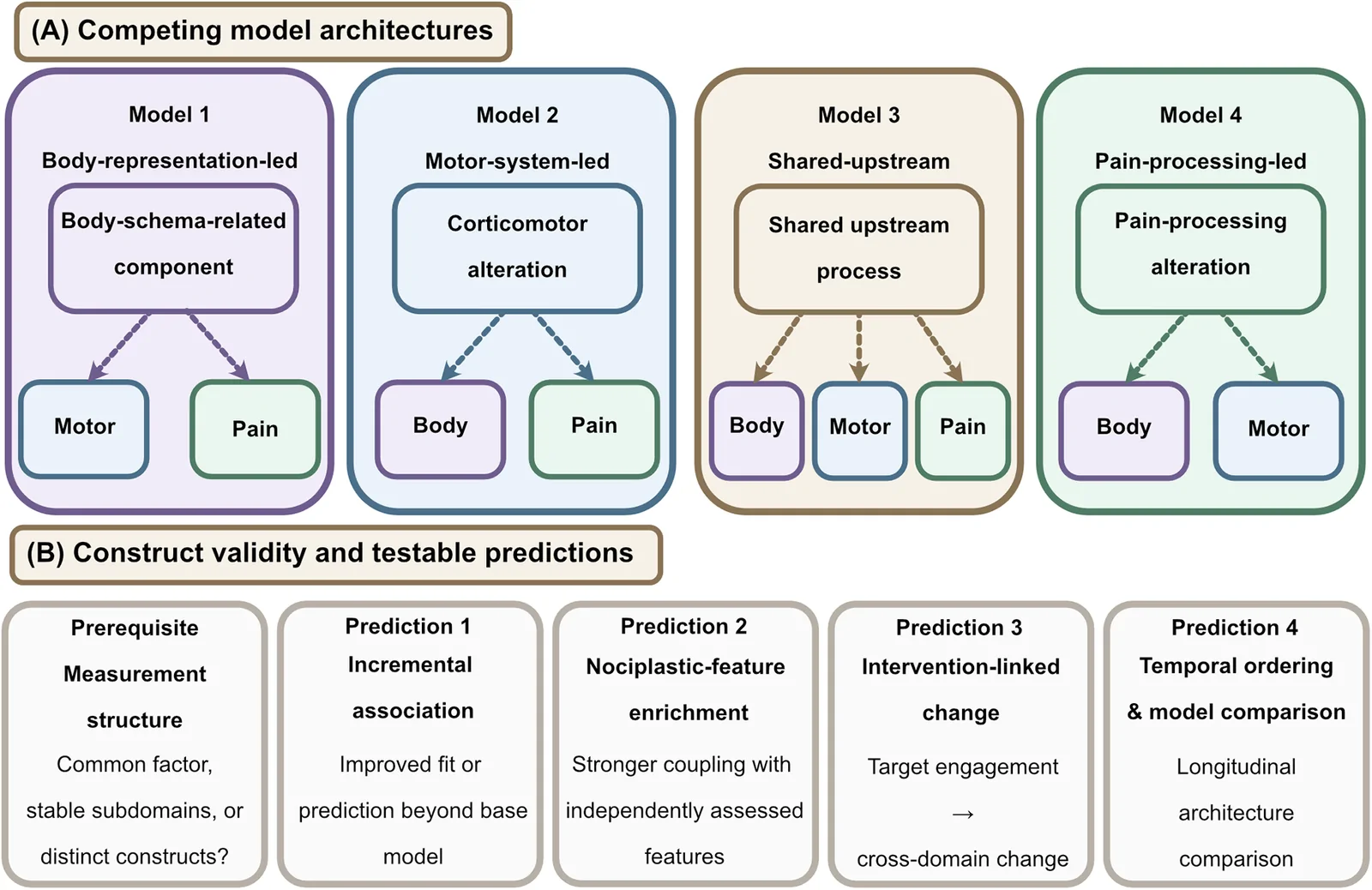
Competing model architectures and testable predictions for the proposed cross-domain framework in chronic non-specific low back pain (CNSLBP). **(A)** Model 1 presents the focal body-representation-led hypothesis, with a candidate body-schema-related sensorimotor component as the organizing node. Models 2–4 instead place corticomotor alteration, a shared upstream process, or altered pain processing in the organizing position. Body, Motor, and Pain denote the body-representation, corticomotor, and pain-processing domains, respectively. Colors identify domains, whereas the outer frame, upper node, and outgoing arrows identify each model's proposed organizing source. Dashed arrows indicate hypothesized ordering rather than established causality. Reciprocal maintenance may occur within any architecture but is omitted for clarity; the diagram does not provide evidence for any model. **(B)** Construct validity is a prerequisite: current indicators may reflect a common factor, stable subdomains, or largely distinct constructs. Prediction 1 tests incremental fit or prediction beyond a prespecified base model; Prediction 2 tests stronger coupling with independently assessed, non-overlapping nociplastic features; Prediction 3 tests whether target engagement is followed by cross-domain change; and Prediction 4 tests temporal ordering through longitudinal comparison of the competing architectures. Failure at any stage narrows or weakens the corresponding claim.

### 6.3 Mutual reinforcement across domains

If the cross-domain coupling proposed in Section 6.2 is present, pain-related fear and threat appraisal could contribute to its maintenance by promoting avoidance, guarded movement, and reduced activity (64). Because movement also samples information from the back and provides opportunities to update body-related internal models (65), a narrower or more stereotyped movement repertoire could change the proprioceptive, cutaneous, and kinesthetic evidence available for recalibrating trunk-state estimates. Those estimates could then continue to shape expectations about movement and its sensory consequences, while ongoing pain and threat-focused attention influence which bodily signals receive priority. Repeated interaction among these processes could allow alterations in body representation, motor regulation, and pain processing to maintain one another.

As a model-derived maintenance hypothesis, reciprocal influence can be incorporated into any of the four organizing architectures shown in Figure 2A: body-representation-led, pain-processing-led, motor-system-led, or shared-upstream. Mutual reinforcement therefore describes how cross-domain relationships might persist once present, while Section 6.4 compares the candidate organizing sources from which those relationships could arise.

### 6.4 Competing explanatory architectures

Although the reciprocal relationships described in Section 6.3 are theoretically plausible, currently available evidence does not yet identify how cross-domain coupling is initially organized. Figure 2A therefore contrasts four possible architectures that could yield a similar pattern of covariation among body-representation-related, corticomotor, and pain-processing measures while assigning a different role to each domain. The focal body-representation-led architecture places miscalibrated trunk-related multisensory and sensorimotor state estimation in a candidate organizing position, from which it could influence motor regulation and the weighting or interpretation of bodily information in pain-related contexts. A pain-processing-led architecture assigns this position to altered pain sensitivity, facilitation, or modulation, with changes in movement and body-related perception developing as adaptations to, or correlates of, the prevailing pain-processing state. Threat appraisal may mediate or modify this pathway. Pain-related fear and avoidance provide one established pathway through which threat appraisal can promote guarded movement and reduced activity (64), while evidence across chronic-pain conditions leaves open the possibility that pain contributes to altered conscious body perception (66). In a motor-system-led architecture, persistent changes in corticomotor regulation, trunk recruitment, or movement diversity are proposed to alter the proprioceptive, tactile, and kinesthetic information sampled during action and thereby the evidence available for updating trunk-state estimates (65). Changes in body-related perception or pain processing would consequently arise within an altered motor and sensory context. Under a shared-upstream architecture, none of the three measured domains occupies the primary organizing position. Threat learning, attention, affective or interoceptive state, habitual activity, distributed neural processes, or systemic influences may generate parallel variation across the domains without requiring direct causal influence among them (64, 65). Psychological and behavioral variables therefore occupy different positions across the candidate architectures. Fear, catastrophizing, distress, expectations, attention, and activity may operate as upstream common influences, mediate or modify a directional pathway, or emerge as consequences of persistent disturbance. Once reciprocal feedback develops, different initiating architectures may produce increasingly similar cross-sectional patterns; reciprocity is therefore treated as a possible maintenance process within each architecture rather than as a fifth initiating model.

The focal hypothesis is relatively narrow, whereas a broader internal-model account would operate at a different explanatory scale. The Fit-for-Purpose model, for example, describes CNSLBP as a state in which relatively stable models of a damaged, fragile, or unhealthy back shape the availability and weighting of sensory, cognitive, behavioral, and neuroimmune information, while movement provides opportunities to sample bodily information and update those models (65). The body-schema-related pathway proposed here can be accommodated within that broader architecture as an action-oriented sensorimotor component, while the broader account also permits relationships among pain, movement, and bodily perception through routes that do not depend on trunk-state estimation. The rationale for examining the narrower component separately lies in target specification. The focal model identifies recalibration of trunk-related multisensory and sensorimotor state estimation as a candidate experimental leverage point whose engagement could, in principle, be distinguished from changes in conscious body perception, beliefs, general task performance, or non-specific treatment response. Its independent contribution would be supported if a valid indicator of this component could be selectively changed and that change preceded or mediated prespecified changes in motor regulation or pain-processing measures. If supported, this would provide a more precisely specified sensorimotor entry point for intervention development within a broader rehabilitation framework. Establishing that role requires the construct-validity and cross-domain tests specified in Section 6.5.

### 6.5 Construct-validity prerequisite and testable predictions

The alternative architectures outlined above lead to a sequence of linked empirical questions. The first concerns what the candidate body-representation measures represent, because the meaning of any subsequent cross-domain association depends on the measurement level at which those measures can be interpreted. Once that level has been established, the framework can be evaluated through incremental association, nociplastic-feature enrichment, intervention-linked change, and temporal model comparison (Figure 2B).

#### 6.5.1 Prerequisite. Construct validity and measurement structure

LRJT accuracy and response time, lumbar 2PDT, and FreBAQ scores capture laterality or motor-imagery-related performance, conscious tactile-spatial acuity, and self-reported back perception, respectively. Their place within the framework therefore depends on whether they reflect a reproducible common factor within the broader altered-body-representation domain, form stable and theoretically interpretable subdomains, or behave largely as distinct constructs. Current evidence does not resolve this structure, and the measures differ in both psychometric support and protocol dependence (30, 32). Before they are combined or used to interpret longitudinal or intervention-linked change, studies should prespecify the relevant protocol and outcome, establish adequate reliability, quantify measurement error, test convergent and discriminant validity, and evaluate responsiveness. Table 1 summarizes the evidence and interpretive boundaries that inform this prerequisite.

**Table 1:** Candidate body-representation-related measures in the proposed framework: observable outputs, measurement status, and interpretive boundaries.

Candidate measure	Observable output	Measurement status	Role and interpretive boundary
Left/right judgment task (LRJT)	Accuracy and response time during trunk laterality judgments; performance may involve implicit motor imagery.	A COSMIN-based review found moderate evidence for known-groups validity, but reliability, measurement error, and convergent validity could not be established from the available studies (30). Across low-back-pain studies, pooled response time was unchanged, whereas lower accuracy was substantially heterogeneous (29).	LRJT can serve as an indirect task-based indicator of action-oriented body representation. Performance may also reflect visual or object-rotation strategies, attention, response selection, and general task performance; an abnormal score does not identify body-schema-related disturbance or a specific neural mechanism in an individual. Interpretation is task conditional and depends on the stimulus set, depicted movement amplitude or complexity, timing, and whether accuracy or response time is analyzed (29, 45, 49).
Lumbar two-point discrimination threshold (2PDT)	Threshold for consciously distinguishing two tactile points over the lumbar region; higher thresholds indicate poorer tactile-spatial acuity.	A COSMIN-based review reported moderate evidence for known-groups validity, whereas reliability, measurement error, and convergent validity could not be established (30).	2PDT can serve as a protocol-specific indicator of conscious lumbar tactile-spatial discrimination. Thresholds depend on test orientation, anatomical site, stimulus and thresholding procedures, repetitions, and examiner administration. Reported associations with selected S1 measures (12, 43) do not make 2PDT a direct cortical-map readout or a neural marker specific to low back pain.
Fremantle Back Awareness Questionnaire (FreBAQ)	Self-reported, consciously experienced disturbance in back perception.	Test–retest reliability was supportive but heterogeneous, and construct validity was rated sufficient on limited-quality evidence. Content validity was inconsistent, structural validity insufficient, internal consistency and measurement error indeterminate, and responsiveness was not evaluated. FreBAQ was classified as COSMIN category B (32).	FreBAQ provides a self-report indicator of conscious back-perception disturbance (13). It does not directly identify disturbance of the action-oriented body-schema-related sensorimotor component or a specific sensorimotor or neural mechanism. Scores also covary with pain, disability, and psychological variables (13).

COSMIN, Consensus-based Standards for the selection of health measurement instruments; S1, primary somatosensory cortex.

The structure supported by these analyses would determine the measurement unit carried into subsequent cross-domain tests. A reproducible common factor would permit factor-level tests, whereas stable subdomains or largely distinct constructs would require the cross-domain hypotheses to be examined separately at those levels. Failure to identify a reproducible common or multidimensional structure would limit aggregate claims about altered body representation while leaving measure-specific hypotheses available for study.

#### 6.5.2 Prediction 1. Incremental cross-domain association

Once the relevant measurement level has been established, the first prediction is that body-representation-related measures will contribute information about corticomotor and pain-processing outcomes beyond variables already known to accompany CNSLBP. For each prespecified outcome, an appropriate base model should therefore be compared with the otherwise identical model after body-representation indicators or validated subdomain scores have been added. Base models should include relevant clinical variables such as pain intensity, disability, symptom duration, and age. Nociplastic-feature, psychological, and behavioral variables should be incorporated according to their prespecified positions, as potential common influences, mediators, modifiers, or outcomes, because their meaning differs across the architectures described in Section 6.4. Replicated improvement in model fit or out-of-sample prediction would support incremental explanatory information. A relationship with only one adjacent domain would support a partial cross-domain pattern, whereas coordinated relationships with both corticomotor and pain-processing measures would provide stronger support for the proposed triadic pattern. Mechanistic independence would still require the intervention-linked and temporal evidence addressed below, and clinical added value would require prospective evidence that the measures improve prognostic or treatment-response prediction, patient stratification, or clinical decision-making.

#### 6.5.3 Prediction 2. Nociplastic-feature enrichment

The model allows the strength of these cross-domain relationships to vary across people with CNSLBP. It therefore predicts that the prespecified associations identified in Prediction 1 should be stronger when independently assessed clinical and psychophysical nociplastic features converge. For each analysis, a measure used as a Prediction 1 outcome should not also contribute to the enrichment definition. Candidate indicators, used only when they do not overlap with the tested outcome, include clinical criteria for possible or probable nociplastic pain (61), pressure pain thresholds measured at symptomatic and remote sites (57), temporal summation (58), conditioned pain modulation (58, 59), and self-reported multisystem symptom burden measured with the Central Sensitization Inventory (63). Temporal summation and conditioned pain modulation should remain separately represented because their association within CLBP is modest (60). The Central Sensitization Inventory should likewise remain a symptom-burden measure and should not independently classify nociplastic pain or establish central sensitization (62). Any cumulative score or latent nociplastic-feature burden would require its own validation. Absence of enrichment would weaken this subgroup-specific prediction while leaving the more general incremental-association hypothesis open.

#### 6.5.4 Prediction 3. Intervention-linked cross-domain change

Incremental association and subgroup enrichment would identify a structured pattern, but the proposed clinical role of the narrower sensorimotor component depends on whether it can be engaged selectively and whether change extends beyond the measure being trained. An intervention intended to recalibrate trunk-related multisensory and sensorimotor state estimation should first produce change in a construct-valid target indicator beyond measurement error and task-practice effects. The focal pathway would gain stronger support if that target change subsequently predicted or mediated prespecified change in motor regulation and/or pain-processing measures after expectancy, attention, activity exposure, and general treatment effects had been considered. A partial precedent comes from a mediation analysis of education and graded sensorimotor retraining, which examined tactile acuity, motor coordination, and back self-perception alongside psychological mediators (67). Because trunk corticomotor measures and psychophysical pain-processing outcomes were not included, that study did not test the three-domain pathway proposed here. Change confined to the trained proxy would indicate task-specific change or local target engagement. Change extending to one adjacent domain would support a partial pathway, while coordinated and temporally ordered change across both adjacent domains would provide stronger evidence for triadic coupling.

#### 6.5.5 Prediction 4. Temporal ordering and model comparison

Where Prediction 3 asks whether deliberate engagement of a selected target can propagate change, Prediction 4 asks how the three domains unfold during the natural development and maintenance of low back pain. Acute-inception cohorts and repeated-measures studies of established CNSLBP should therefore assess body-representation-related, corticomotor, and pain-processing measures concurrently. The four organizing architectures, body-representation-led, motor-system-led, pain-processing-led, and shared-upstream, can then be compared in longitudinal models that account for prior levels of each domain and allow reciprocal effects to emerge. Under the focal architecture, early change in construct-valid indicators of trunk-related sensorimotor state estimation should predict later motor or pain-processing variation beyond the prior values of those outcomes. Prospective evidence that smaller S1- and S2-related somatosensory evoked-potential responses to non-noxious lumbar stimulation during acute low back pain were associated with pain persisting at six months provides a precedent for this temporal design (68). Because those recordings followed pain onset and body-representation-related measures were not collected, the study does not determine ordering among the three proposed domains or distinguish a pre-existing susceptibility from an early pain-related change. Better replicated fit of a pain-processing-led, motor-system-led, or shared-upstream architecture would weaken body-representation-led priority. Reciprocal effects without a reproducible body-representation-led temporal asymmetry would support cross-domain coupling while weakening directional priority.

The interpretation of a null result depends on where this sequence breaks down. An unstable measurement structure would limit aggregate construct claims; adequate measurement structure without incremental association would weaken the proposed cross-domain contribution; absence of enrichment would narrow the subgroup hypothesis; and target engagement without subsequent cross-domain change would weaken the proposed sensorimotor pathway. Consistent longitudinal support for an alternative architecture would require revision of the proposed directional ordering while leaving measure-specific findings and possible cross-domain coupling intact.

## 7 Discussion and conclusion

The central contribution of the present framework is to convert a broad family of body-representation-related observations into a more specific question about cross-domain organization. Altered body representation encompasses distinguishable tactile-spatial, laterality-related, consciously experienced perceptual, and movement-perception phenomena, whereas the focal body-schema-related hypothesis concerns an action-oriented process involved in estimating trunk configuration and movement. A separate explanatory role for this narrower component would require a construct-valid indicator of trunk-state estimation, incremental information beyond established clinical and psychological variables, and temporally or perturbationally ordered relationships with corticomotor and pain-processing outcomes. Existing studies establish the empirical relevance of several measure-specific phenomena, but they do not yet establish a coherent trunk-state-estimation construct, clinical added value, or causal priority. The body-representation-led architecture is therefore treated as one candidate organization alongside pain-processing-led, motor-system-led, and shared-upstream accounts, with reciprocal processes potentially contributing to maintenance within each.

Preserved mechanoreceptive somatotopy narrows the level at which the focal hypothesis can operate. During passive thoracolumbar vibrotactile stimulation, segmental representational structure was preserved across S1, M1, and superior parietal cortex in CLBP, without corrected group differences or stable associations with pain or FreBAQ scores, despite greater self-reported back-perception disturbance in the patient group (34). This pattern argues against degradation of the local spatial geometry of mechanoreceptive input as a necessary explanation of altered back perception. The foundational magnetoencephalography study used painful intracutaneous stimulation and reported enhanced evoked responses and a medial shift of the estimated S1 source in a small sample meeting broader chronic-back-pain criteria (9). Because the studies differ in stimulus modality, imaging method, and representational property, the later finding provides an important boundary condition but does not constitute a direct replication of the earlier result.

The resulting theoretical question concerns the dynamic use and updating of body-related information. Body schema is an action-oriented representation that supplies bodily information for action programming, prediction, and sensory feedback (27). Predictive coding and active inference provide a general vocabulary for describing how state estimates may be updated through precision-weighted sensory discrepancies and used to generate proprioceptive predictions for action (23, 33). Applied conditionally to CNSLBP, spatially organized tactile input could be weighted, combined with visual and proprioceptive information, or deployed differently when estimating trunk configuration and movement. This extension is model-derived: neither the preserved-somatotopy finding nor the general computational literature demonstrates altered trunk-state updating in CNSLBP. It nevertheless yields a discriminating prediction. Passive segmental topography may remain intact while cross-modal recalibration, trial-by-trial updating of trunk-state estimates, or the use of those estimates in anticipatory motor regulation is altered. Testing this dissociation requires concurrent assessment of passive representational structure, dynamic state-estimation processes, and the proposed motor and pain-processing outcomes.

This dynamic formulation also changes how heterogeneity should be interpreted. A task- and context-dependent operation need not produce equivalent findings in 2PDT, LRJT, FreBAQ, passive cortical mapping, and corticomotor measures. Task-dependent dissociation could reflect multidimensional or state-dependent body representation, while non-specific task demands, protocol variation, and measurement error could generate a similar pattern. Current evidence cannot distinguish these possibilities: trunk LRJT accuracy effects are substantially heterogeneous (29), FreBAQ structural validity is insufficient and responsiveness remains unassessed (32), corticomotor findings vary according to target muscle, outcome, and protocol, with only selected paraspinal findings remaining robust in meta-analytic sensitivity analyses (18, 19, 52, 56), and group differences in temporal summation and conditioned pain modulation are small or inconsistent across heterogeneous studies (58, 59). A reproducible within-person and cross-domain profile under prespecified task and phenotypic conditions is therefore more informative than abnormality on any single measure. No such subgroup profile has yet been established, and candidate subgroup or task conditions require prospective definition and replication.

At a broader explanatory level, the focal hypothesis can be situated within existing models of CNSLBP. The Fit-for-Purpose model describes a multilevel information problem in which beliefs about back fragility, attention, sensory evidence, movement behavior, tissue capacity, and broader biological processes may reinforce one another (65). Fear-related threat appraisal provides one established route through which avoidance and guarded activity can alter movement behavior (64). A trunk-state-estimation pathway could be accommodated within that wider architecture as an action-oriented sensorimotor component, while pain and disability could also change through pathways that do not depend on it. The distinctive contribution of the present proposal is its finer level of target specification: the candidate component can be operationalized, selectively perturbed, and tested for propagation to motor and pain-processing outcomes. Psychological and behavioral variables may act upstream of, interact with, mediate, or follow this pathway; their causal position should therefore be specified for each architecture rather than handled as a uniform adjustment set.

Clinical translation follows from this level of target specification. The proposed experimental target is recalibration of trunk-related multisensory and sensorimotor state estimation. Existing paradigms provide partial examples of relevant perturbations, including manipulation of visual feedback about active trunk movement (50) and sensory-discrimination or motor-imagery approaches developed mainly in other chronic-pain conditions (69). Visual, tactile, proprioceptive, and movement-based approaches would belong to the same mechanistic class only when they are designed to change the information used to estimate trunk state and demonstrate engagement of that process. Improvement confined to a practiced task would indicate local learning or responsiveness. Evidence of recalibration would require change beyond measurement error and practice effects, generalization to untrained body-state judgments or movement contexts, and temporally ordered change in prespecified corticomotor and/or psychophysical pain-processing outcomes. This functional definition allows different intervention modalities to test the same candidate process without assuming that their clinical labels imply a common mechanism.

Current measures do not yet define this target at the level of an individual patient. LRJT, 2PDT, and FreBAQ cannot diagnose body-schema disturbance; the Central Sensitization Inventory alone cannot establish nociplastic pain; and neither transcranial magnetic stimulation nor conditioned pain modulation measures have validated treatment-selection roles within this framework (30, 32, 62). Existing CNSLBP intervention evidence provides only a partial precedent. Multicomponent education and graded sensorimotor retraining produced a small pain benefit compared with sham procedures and attention control (70). In the subsequent mediation analysis, the largest estimated indirect effects involved back beliefs, catastrophizing, and pain self-efficacy; a smaller indirect effect was estimated through back self-perception, whereas tactile acuity was not supported as a mediator (67). Because trunk corticomotor and psychophysical pain-processing measures were not included, the proposed three-domain pathway remains untested. These findings show why clinical improvement, mediation through broader psychological or perceptual processes, and engagement of the narrower trunk-state target must remain distinguishable claims.

However, the evidentiary limitations remain substantial. Direct independent replication is limited because later studies have frequently examined adjacent tasks, cortical properties, or patient populations rather than reproducing the original design. Conceptual convergence across tactile, structural, connectivity, and perceptual findings supports continued investigation, but it cannot establish marker stability or replication of a specific remapping result. The targeted, non-systematic method used in this article also precludes estimation of the prevalence and consistency of the proposed phenomena or quantitative evaluation of publication bias, small-study effects, and selective outcome reporting. Positive and mechanistically interpretable findings may consequently be overrepresented. The present synthesis therefore serves hypothesis construction and model specification, while the proposed cross-domain relationships remain to be tested directly.

Under this formulation, altered body representation remains a broad and heterogeneous measurement domain, while body-schema-related trunk-state estimation remains a narrower working hypothesis about the organization of sensorimotor, motor, and pain-processing relationships. Support for an independent role would require construct-valid target engagement that generalizes beyond a trained proxy and precedes or mediates prespecified cross-domain change. Preserved dynamic state-estimation processes, change confined to the targeted task, failure of cross-domain relationships to replicate, or better fit of pain-processing-led, motor-system-led, or shared-upstream architectures would require corresponding revision of the focal claim. The proposed contribution is therefore a candidate experimental leverage point within a broader rehabilitation landscape; its mechanistic independence and clinical stratification value remain to be established.

## Data Availability

The original contributions presented in the study are included in the article/supplementary material, further inquiries can be directed to the corresponding author.
